# Bacterial contamination of inanimate surfaces and equipment in the intensive care unit

**DOI:** 10.1186/s40560-015-0120-5

**Published:** 2015-12-10

**Authors:** Vincenzo Russotto, Andrea Cortegiani, Santi Maurizio Raineri, Antonino Giarratano

**Affiliations:** Department of Biopathology and Medical Biotechnologies (DIBIMED), Section of Anaesthesia, Analgesia, Intensive Care and Emergency, University Hospital Paolo Giaccone, University of Palermo, Via del Vespro 129, 90127 Palermo, Italy

**Keywords:** Equipment contamination, Bacterial contamination, ICU, Multidrug resistance

## Abstract

Intensive care unit (ICU)-acquired infections are a challenging health problem worldwide, especially when caused by multidrug-resistant (MDR) pathogens. In ICUs, inanimate surfaces and equipment (e.g., bedrails, stethoscopes, medical charts, ultrasound machine) may be contaminated by bacteria, including MDR isolates. Cross-transmission of microorganisms from inanimate surfaces may have a significant role for ICU-acquired colonization and infections. Contamination may result from healthcare workers’ hands or by direct patient shedding of bacteria which are able to survive up to several months on dry surfaces. A higher environmental contamination has been reported around infected patients than around patients who are only colonized and, in this last group, a correlation has been observed between frequency of environmental contamination and culture-positive body sites. Healthcare workers not only contaminate their hands after direct patient contact but also after touching inanimate surfaces and equipment in the patient zone (the patient and his/her immediate surroundings). Inadequate hand hygiene before and after entering a patient zone may result in cross-transmission of pathogens and patient colonization or infection. A number of equipment items and commonly used objects in ICU carry bacteria which, in most cases, show the same antibiotic susceptibility profiles of those isolated from patients. The aim of this review is to provide an updated evidence about contamination of inanimate surfaces and equipment in ICU in light of the concept of patient zone and the possible implications for bacterial pathogen cross-transmission to critically ill patients.

## Introduction

Intensive care unit (ICU)-acquired infections are a major cause of morbidity and mortality worldwide [1]. Infections caused by multidrug-resistant (MDR) bacteria are a worrisome healthcare problem and a daily challenge for the clinician dealing with critically ill patients [2, 3]. Contamination of inanimate surfaces in ICU has been identified in outbreaks [4–6] and cross-transmission of pathogens among critically ill patients [7, 8]. Contamination may occur either by transfer of microorganisms contaminating healthworkers’ hands or direct patient shedding of microorganisms in the immediate environment of a patient’s bed [9]. MDR bacteria have been reported as contaminating microorganisms of surfaces, commonly used medical equipment and high-contact communal surfaces (e.g., telephones, keyboard, medical charts) in ICU [10–13]. It has been reported that both Gram-positive and Gram-negative bacteria are able to survive up to months on dry inanimate surfaces, with longer persistence under humid and lower-temperature conditions [14]. Factors that may affect the transfer of microorganisms from one surface to another and cross-contamination rates are type of organisms, source and destination surfaces, humidity level, and size of inoculum [15, 16]. However, other factors playing a role in contamination and cross-transmission rate in the ICU may include hand hygiene compliance, nurse-staffing levels, frequency/number of colonized or infected patients, ICU structural features (e.g., single-bed or multi-bed ICU rooms) and adoption of antibiotic stewardship programs [17, 18]. The issue of environmental contamination may pose an even greater challenge in the ICU, where patients are critically ill, with several risk factors for nosocomial infections [19], and the highest standard measures for infection prevention cannot always be addressed due to impelling, life-threatening conditions. Moreover, the nearby environment of ICU beds is crowded by equipment for monitoring and support, with many hand-touch sites, requiring sophisticated and specific cleaning procedures [20]. Identifying which sites are more frequently contaminated and what the most commonly identified contaminants are may play a major role for infection control practices and promotion of new interventions [16]. Environmental contamination by fungi and viruses has been also described in ICU [21, 22]. However, in this review, we focused on bacterial contamination. The aim is to provide an updated evidence on contamination of inanimate surfaces, equipment, and high-contact communal surfaces in ICU, focusing on most commonly isolated bacteria, the role of contamination for ICU-acquired colonization and infection, and possible implications for care of ICU patients.

## Review

### Inanimate surface contamination and ICU-acquired colonization and infections: the concepts of *patient zone* and *healthcare area*

**Fig. 1:**
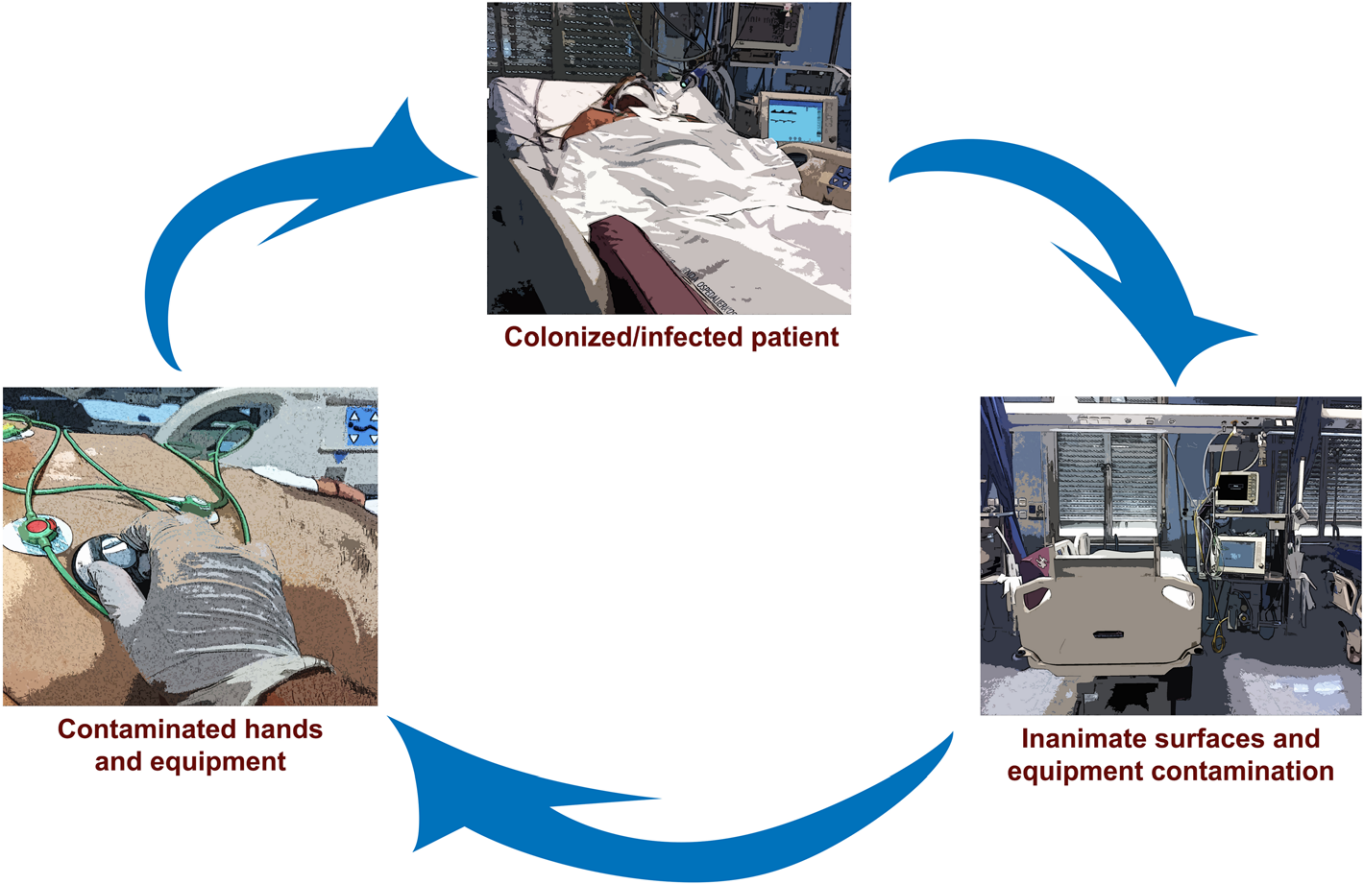
Role of ICU environmental contamination for patient colonization/infection [15]. Each *stage* may represent the starting point of a process that may follow either a clockwise or counterclockwise direction

A growing body of evidence supports the contribution of inanimate surface and equipment contamination for transmission of pathogens to ICU patients. Healthcare workers’ hands are the major vector of cross-transmission of pathogens, with an estimated 20 to 40 % of nosocomial infections arising from cross-infections via healthcare personnel hands [11, 23]. Bacterial contamination of caregivers’ hands increases linearly over time, with a progressively higher grade of contamination with longer duration of care [24]. It commonly occurs after direct patient contact. However, healthcare workers may contaminate their hands after contact with inanimate surfaces surrounding a patient’s bed (e.g., ground, bedrails, emergency carts, and trolleys) or after usage of high-contact equipment items and objects (e.g., stethoscopes, monitors, ventilators, phones, medical charts) [9, 25, 26]. Evidence from observational studies identifies colonized and infected patients as a reservoir for environmental contamination [16, 27]. Frequently touched surfaces and objects in the immediate vicinity of patients are more frequently and heavily contaminated [9]. The concepts of *patient zone* and *healthcare area* have been proposed as a user-centered, geographically related model designed to improve hand hygiene compliance by healthcare personnel during their daily workflow [28]. The patient zone encompasses the patient and his/her immediate surroundings. Inanimate surfaces in the patient zone are rapidly contaminated by microorganisms after direct patient shedding of bacteria, or indirectly due to high-frequency interactions between healthworkers’ hands and high-touch surfaces (e.g., monitors, ventilator buttons, bedrails), in the patient zone. The healthcare area includes all surfaces outside a given patient zone, namely the healthcare facility environment and other patient zones. Healthcare area may be contaminated by microorganisms from different patient zones [28]. Healthcare workers, crossing different patient zones, may be responsible for cross-transmission and further environment contamination in case of poor hand hygiene compliance [16, 26, 28]. Inanimate surfaces and equipment in the patient zone (e.g., bedrails, ventilator surfaces) should be regularly cleaned due to the high and rapid contamination. Equipment in the healthcare area may be introduced into a patient zone for monitoring or therapeutic purposes (e.g., ultrasound and portable radiograph equipment) and should be cleaned before being brought in the patient zone and after being taken out from it [29]. In a randomized cross-over study, recontamination of high-contact surfaces in ICUs occurred after 4 h from standard cleaning measures [30]. Notably, the rate of healthcares’ hand or glove contamination after surface contact is comparable to that observed after patient direct contact [9]. Figure 1 illustrates the role of contamination of surfaces and equipment in ICU. The figure should be read as a circle process, and each stage may be considered the starting point. Possible outcomes of this process are cross-transmission of microorganisms, leading to colonization or infection of new patients (belonging to two different patient zones), and healthcare area further contamination. Notably, colonization has been identified as a risk factor for subsequent infection caused by different bacterial species in ICU [19, 27]. In this regard, cross-transmission, leading to patients colonization, should be considered a negative outcome per se [19, 31]. Moreover, in different outbreak reports [4] and observational studies [7, 8, 12], MDR isolates were responsible for environment contamination [32]. These data raise concern about a potential role of contamination as a reservoir for resistant species, their selection and subsequent development of ICU-acquired colonization and infections. For these reasons, the issue of environmental contamination has been included in a recently published bundle of recommendations aiming to reduce the incidence of ICU-acquired infections caused by MDR pathogens [17]. However, further studies are needed to elucidate the contribution of inanimate surfaces and equipment contamination to relevant patient outcomes (e.g., mortality, ICU length of stay). A higher shedding of pathogens has been observed from infected patients than from those who are only colonized, with a correlation between frequency of contamination and number of culture-positive body sites [18, 32]. Moreover, a higher environmental contamination has been observed around patients with diarrhea [33, 34]. Bacteria shed from patients are able to survive up to months on dry inanimate surfaces with a concentration sufficient to cause transmission in most cases. When we analyze the association between environment and patient transmission of microorganisms, the temporal relationship between contamination and transmission should be addressed, along with the presence of potential confounders (e.g., the quality of environmental cleaning and hand hygiene) and the reduced incidence of cross-transmission when control measures have been undertaken [16]. The molecular identification of bacterial strains responsible for cross-transmission and/or nosocomial infection has provided useful insights about the role of environmental contamination [10]. Notably, patients may be colonized by isolates different from those detected on surfaces or medical equipment and may result from endogenous flora spread. The same genetic profile of isolates has matched, instead, when environmental contamination has been claimed to play a role on patient colonization or infections [23]. The role of inanimate surface contamination for acquisition of nosocomial pathogens has been further highlighted by studies investigating the role of residual contamination after post-discharge cleaning (i.e., terminal cleaning) for colonization or infection of patients occupying rooms of previously infected patients. In a retrospective study performed in eight adult ICUs at a tertiary care hospital, investigators assessed the risk of acquiring methicillin-resistant *Staphylococcus aureus* (MRSA) and vancomicin-resistant enterococci (VRE) from prior room occupants. Patients were screened on admission and weekly for MRSA and VRE carriage. Patients occupying rooms of carriers showed a significantly higher risk of acquisition of MRSA (odds ratio, OR 1.4, 95 % confidence interval, CI 1.0–1.9) and VRE (OR 1.6, 95 % CI 1.2–2.2). This increased risk was still observed after correction for other variables (e.g., age, comorbidities, pre-ICU length of stay) [7]. Notably, in all participating ICUs, terminal room cleaning was performed according to recommended standards, with additional precautions adopted in adherence to local protocols. In a prospective cohort study, the risk of acquiring pathogens from prior room occupants was investigated for MDR Gram-negative bacilli. Carriage of MDR bacteria by prior room occupants was the most important risk factor for ICU-acquired *Pseudonas aeruginosa* (OR 2.3, 95 % CI 1.2–4.3) and the second most important independent risk factor for *Acinetobacter baumannii* acquisition (OR 4.2, 95 % CI 2.0–8.8), after mechanical ventilation [8]. Viable MDR bacteria have been isolated in biofilm on surfaces and furnishings sampled after terminal cleaning in a 16-bed ICU [35]. Biofilm may enhance bacterial survival capacity on dry surfaces and may confer resistance against physical and chemical agents. Indeed, viable bacteria within biofilms are up to 1500 times more resistant to biocides than those growing in a liquid culture [36]. It may be hypothesized that biofilm formation may contribute to the observed residual contamination after terminal cleaning procedures currently in use. These results may highlight the lack of full eradication of contaminating pathogens after currently recommended standard for terminal room cleaning, although this inefficiency may be attributed to several factors involved in the process (e.g., type of product, sufficient time contact, shortcomings in the procedure). In summary, the patient zone of colonized or infected patients is heavily contaminated by bacteria, including MDR species. Healthcare workers’ hands have a major role in environmental contamination, along with direct patient shedding [26]. Inanimate surface contamination serves as a reservoir for patient cross-transmission of bacteria and may contribute to patients colonization and, in some circumstances, infection [23]. In the absence of appropriate hand hygiene and other infection control measures, colonized and infected patients are the starting point of a new vicious circle [16, 28].

### Evidence of equipment and commonly-used-object contamination in ICU

ICU patients are exposed daily to a number of monitoring devices and support equipment. Invasive devices are defined as those interrupting skin and mucosal integrity or being in direct contact with patient’s blood and mucosa (e.g., endotracheal tubes, central venous catheters). The association between invasive devices and nosocomial infections has been clearly established (e.g., ventilator-associated pneumonia, catheter-associated bloodstream infections) [23]. A number of reports, observational and infection control studies, highlighted the role of non-invasive ICU devices as a potential source of hospital-acquired infections [4, 37, 38]. In the majority of cases, contamination has involved electrical equipment [6, 39, 40] or difficult-to-clean items due to irregular/hidden surfaces or lacking disinfection guidelines [40]. To date, evidence of direct contribution of environmental contamination for nosocomial infection is uncertain. In the following paragraphs, we reviewed the evidence about contamination of some, commonly used, ICU equipment items. The most commonly identified pathogens are summarized in Table 1. The following paragraphs aim to provide examples of equipment contamination in ICU. Ineffective cleaning procedures and infection control measures may similarly be responsible for contamination of different equipment items and objects in the ICU environment. It is beyond the scope of this review to provide details about cleaning and disinfection procedures in ICU. Different reviews on this topic have been recently published [41, 42].

**Table 1:** Examples of items/equipment with reported contaminating bacteria in ICU

Contaminated item/equipment in ICU	Microorganisms	References
ECG leads	VRE	Falk et al. (2000) [4]
	Coagulase-negative staphylococci, *P. aeruginosa*	Lestari et al. (2013) [40]
Blood pressure cuffs	*S. aureus* (MRSA)	Matsuo et al. (2013) [66]
Ventilator (e.g., buttons, circuits)	*S. aureus* *P. aeruginosa*	Sui et al. (2012) [46]
Suction system switches	*S. aureus* *P. aeruginosa*	Sui et al. (2012) [46]
Medical charts	Coagulase-negative staphylococci*,* *A. baumannii* *K. pneumoniae*	Teng et al. (2009) [38]
Portable radiograph equipment	*S. aureus* (MRSA) VRE *A. baumannii* *K. pneumoniae* *P. aeruginosa*	Levin et al. (2009) [12]
Ultrasound machine	*S. aureus* (MRSA, MSSA) Coagulase-negative staphylococci *P. aeruginosa* A. baumannii *Corinenebacterium spp.* *Bacillus spp.*	Shokoohi et al. (2015) [20] Koibuchi et al. (2013) [57]
Bed rails	*A. baumannii*	Catalano et al*. (1999)* [67]
Stethoscopes	*S. aureus* *A. baumannii*	Whittington et al. (2009) [45]
White coats/scrubs	*A. baumannii*	Munoz-Price et al. (2012) [68]
Telephone/cell phones	*A. baumannii*	Borer et al. (2005)
	Coagulase-negative staphylococci *S. aureus* Non-fermenting Gram-negative bacteria	Ulger et al. (2009) [13]
Computer keyboards	Coagulase-negative staphylococci Non-fermenting Gram-negative bacteria	Rutala et al. (2006) [69]
Handwashing sink	*Klebsiella spp.*	Roux et al. (2013) [70]

*MRSA* methicillin-resistant *Staphylococcus aureus*, *MSSA* methicillin-sensitive *Staphylococcus aureus*, *VRE* vancomycin-resistant enterococci

#### Electrocardiography lead wires

Manually cleaned, reusable electrocardiography (ECG) lead wires are widely used in ICU. They are placed on direct contact with intact skin, but they may take close proximity with wounds, intravenous lines, surgical dressings, and injured areas. Contamination of ECG lead wires has been reported during an outbreak of VRE in a burn unit [4], but it has also been assessed by observational studies in which ECG lead wires have been sampled for bacterial contamination [40, 43]. Notably, ECG lead wires were cleaned and ready to use for the following patient before being sampled. ICU lead wires have been reported to be heavily contaminated with a proportion of nosocomial pathogens ranging from approximately 20 [40] to 45 % [43] of total samples. Coagulase-negative staphylococci were the leading Gram-positive bacteria identified, whereas *P. aeruginosa* was the most commonly identified Gram-negative species [40]. Use of disposable ECG lead wires has been claimed as a potential measure to reduce cross-transmission [44].

#### Stethoscopes

Whittington et al. [45] investigated the contamination of both bedside and ICU staff stethoscopes. Both diaphragms and earpieces of sampled stethoscopes used in ICU were heavily contaminated by bacteria (diaphragms; bedside stethoscopes 95 %, personal stethoscopes 67 %; earpieces; bedside stethoscopes 75 %, personal stethoscopes 100 %). Potential pathogenic bacteria were isolated from diaphragms of 14 % of bedside and 8 % of personal stethoscopes. Earpieces carried pathogenic bacteria in 21 and 23 % of bedside and personal stethoscopes, respectively. *S. aureus* was the leading Gram-positive pathogenic species including two MRSA isolates. *Acinetobacter spp*. were the leading Gram-negative pathogenic bacteria, including one isolate of *A. baumannii* resistant to all tested antimicrobials except colistin. Participants were asked to clean stethoscopes according to their preferred method, with alcohol swabs resulting in the leading adopted method. After cleaning, 2 % of diaphragms and 7 % of earpieces were still contaminated. When anonimously answered, compliance with cleaning procedures of stethoscopes was higher among nurses (91 % of those interviewed cleaned them after every use) compared with doctors (only 30 % of those interviewed cleaned them after every use) [45].

#### Surfaces of mechanical ventilators

Sui et al. [46] investigated the bacterial contamination of surfaces of mechanical ventilator systems in a 15-bed respiratory center. Swab sampling not only involved faceplates, ventilator plates, and handrails but also the Y-pieces and water trap surfaces of the breathing circuits. Total bacterial contamination ranged from 70.6 to 100 %. *S. aureus* and *P. aeruginosa* were specifically searched. The highest contamination rate for *S. aureus* was observed on Y-pieces (86.7 %) followed by handrails (64.7 %). The highest contamination by *P. aeruginosa* was reported for water trap surfaces, with no positive cultures for mechanical ventilator surfaces. Contamination rate increased over time, with the highest contamination rate observed after 8 h from the initial surface disinfection. Notably, *P. aeruginosa* contamination electively involved the breathing circuit and persisted, especially on water trap surfaces, following 75 % alcohol treatment. Contact with ground surface by water traps may explain this observation [46].

#### Portable radiograph equipment

Levin et al. [12] investigated the activity of radiograph technicians, focusing on adoption of infection control measures and degree of portable radiograph equipment contamination. They performed a 3-phase study, consisting on an observational phase (assessment of baseline adoption of infection control measures), an intervention phase (notification of contamination results and educational interventions), and a follow-up phase. Susceptible Gram-positive bacteria were detected in 9 % of culture sets, whereas susceptible Gram-negative bacteria were isolated in 45 % of sets. Resistant Gram-negative bacteria (*A. baumannii*, *K. pneumoniae*, *P. aeruginosa*, *Stenotrophomonas maltophilia*) were detected in 39 % of cultures, and a VRE isolate was cultured on one occasion (3 %). Notably, when a resistant Gram-negative species was cultured from the radiograph equipment, the same species was almost always isolated in surveillance or clinical cultures of at least one ICU patient. During the intervention period, promotion of infection control measures resulted in a significant reduction of radiograph equipment contamination, with a decrease of both Gram-positive- and Gram-negative-resistant strains. Radiograph equipment may represent a reservoir for bacteria, including MDR species, in ICU. Equipment and technicians may cross different patient zones during a day, with significant contribution to patients cross-transmission of pathogens when inadequate hygiene measures are undertaken before entering a patient zone. Educational interventions may increase awareness of this potential risk, and radiograph technicians should be involved in infection control programs.

#### Ultrasound equipment

The use of point-of-care ultrasound (US) has greatly increased as part of diagnosis and management of critically ill patients in both the ICU and emergency department. Moreover, several sterile invasive maneuvers are now performed under US guide (e.g., insertion of central venous line, arterial line), posing issues about decontamination and covering of the equipment. All the elements of the ultrasound machine may be contaminated by microorganisms, including probes, keyboards, cords, control settings, gel, and gel bottles [20, 47, 48]. US machines are usually used on several patients, many times per day. Although probes may be disinfected after each use or covered by sterile sheaths, it is unlikely that the entire device is disinfected after every scan [20]. Thus, the devices could remain contaminated passing microorganisms from one patient to an operators’ hands and to other patients [49]. Most of the evidence about US machine contamination came from a study not specifically addressing echo in ICU (e.g., Emergency Department US machines, echo machines for regional anesthesia, whole hospital US equipment) [20, 50–54]. Several studies have demonstrated contamination of elements of echo machines by many types of pathogens, including both MRSA [47, 52] and methicillin-sensible *S. aureus* [50] (most common), coagulase-negative staphylococci [55], *P. aeruginosa* [50], *Corynebacterium spp.* [56], *Acinetobacter spp.* [52], *Bacillus spp.* [57]. Notably, most of the studies collected samples from US machines during normal daily activities, including disinfection according to local protocols. There is evidence of an outbreak by extended spectrum beta-lactamase *K. pneumoniae* originating from contaminated ultrasound-coupling gel [5] and an outbreak of MDR *P. aeruginosa* caused by contaminated transesophageal echocardiography equipment [6]. It has also been demonstrated that, with routine usage, bacterial growth on US machines increases over time from an initial cleaning [53]. The available evidence describes the fact that US cleaning is frequently suboptimal. Manual cleaning is essential to eliminate potentially contaminated gel and other material residuals [20]. It may also be considered that the widespread alcohol-based disinfectants should not be used for disinfection of echo transducers due to the potential damage occurring to the rubber head transducers [58]. It could be recommended to follow available guidelines and manufacture’s recommendation for cleaning procedures, according to the type of usage (i.e., intact skin, wounds, contact with blood, purulent material, MDR-carrying patients) [20]. Clinicians should be aware of the importance to clean not only the probes but also all the other elements of US machines after each use inside a patient zone to reduce the risk of cross-contamination.

#### Medical charts

Medical charts are prone to surface contamination since they are handled by physicians, nurses, and other medical staff several times a day, and they are used for case notes after patient contact for physical examination or invasive procedures. Medical charts may be transferred from one ward to another and may be placed on already contaminated surfaces (e.g., beds, carts). Different studies investigated the contamination of outer surfaces of medical charts in ICU, with an observed contamination rate as high as 80–90 % [38, 59, 60]. In a recently published study, risk of pathogen contamination was two to fourfold higher in ICU compared with general ward. A higher incidence of contamination by MRSA was also reported [60]. Teng et al. [38] investigated contamination of medical charts in a surgical ICU in Taiwan. Ninety percent of sampled medical charts were contaminated. The leading isolated Gram-positive bacteria were coagulase-negative staphylococci, whereas *A. baumannii* and *K. pneumoniae* were the most commonly isolated Gram-negative bacteria. *A. baumannii* was isolated from the corresponding patients in four out of nine contaminated charts, whereas *K. pneumoniae* in two out of three corresponding patients [61]. Notably, antimicrobial susceptibility profiles of isolated bacteria were similar to those from pathogens responsible for patient colonization or infection. Given the similar use of medical charts between general wards and ICUs, it may be hypothesized that their increased risk of contamination in ICU may be due to higher patient shedding of bacteria and environmental contamination. Strict adherence to hand hygiene protocols is advocated before and after medical chart handling [60].

#### Mobile phones

Mobile phones are the most commonly used non-medical portable electronic devices in ICU. They are not only used for communication but also for web consultation and use of applications for patient care (e.g., calculation of infusion doses, electrolytes correction formulas). Unlike fixed phones, of which contamination was also reported [62], mobile phones are often used in close proximity to patients and inside patient zones. A number of reports and observational studies have highlighted the heavy contamination of mobile phones by bacteria, including MDR [63]. In different studies, mobile phone specimens were associated with sampling from the owner’s dominant hand, showing a high degree of correspondence between isolated bacteria [13, 63]. In a study aiming to assess contamination of mobile phones of healthcare workers in operating rooms and ICUs, the rate of bacterial contamination was 94.5 %, with one bacterial species isolated in approxymately 50 % of cases and two or more species detected in about 45 % of total samples [13]. Coagulase-negative staphylococci were the most frequent isolates among Gram-positive bacteria, followed by *S. aureus*. Non-fermenting species were the leading Gram-negative bacteria [13]. In a study performed in Israel, *A. baumannii* has been recovered from mobile phones and corresponding hands. One clone was responsible for patient colonization [37]. Hand contamination after mobile phone-use occurs rapidly; a 1 min call was responsible for 95 % positive samples of previously disinfected hands, in a study assessing the extent of mobile phone contamination among anesthesiologists [39].

### Assessment of environmental contamination: objective monitoring systems

As evidence of the role of environmental contamination for cross-transmission increases, the need for objective monitoring of the cleaning process has emerged, especially in ICU. Objective assessment provides immediate feedback and opportunities to improve hygiene procedures and educational intervention for cleaning staff and healthcare workers. In 2010, the Centers for Disease Control and Prevention (CDC) developed a tool kit providing guidance for development of a program to improve environmental hygiene [64]. Five objective monitoring methods of environmental hygiene were included in the CDC tool kit: (1) direct practice observation of staff performance and compliance with protocols; (2) swab and (3) agar slide cultures, providing a quantitative assessment of viable microbial contamination; (4) fluorescent markers (gel, powder, lotion) used to mark high-touch surfaces; (5) adenosine triphosphate (ATP) bioliminesence, which detects the total amount of both microbial (from either viable or non-viable microorganisms) and non-microbial ATP. When incorporated in programs to improve environmental cleaning, objective monitoring of procedures contributed to significantly reduce the patient zone contamination [29, 65]. A full description of current cleaning technologies and environmental contamination-monitoring systems is beyond the aim of this review, but it has been specifically addressed by recently published reviews and guidelines [29, 41, 64].

## Conclusions

Inanimate surfaces and equipment in ICU are heavily contaminated by bacteria, including MDR species. Bacterial contamination may contribute to ICU-acquired colonization or infection, but further studies are needed to evaluate this correlation. Clinicians and researchers should be aware of the risk of cross-transmission of pathogens from inanimate surfaces in order to adopt appropriate infection control measures.

## Abbreviations

ATP: adenosine triphosphate
CDC: Centers for Disease Control and Prevention
ECG: electrocardiography
ICU: intensive care unit
MDR: multidrug-resistant
MRSA: methicillin-resistant *Staphylococcus aureus*
MSSA: methicillin-sensitive *Staphylococcus aureus*
OR: odds ratio
US: ultrasound
VRE: vancomycin-resistant enterococci
